# The Accuracies of Diagnosing Pancreas Divisum by Magnetic Resonance Cholangiopancreatography and Endoscopic Ultrasound: A Systematic Review and Meta-analysis

**DOI:** 10.1038/srep35389

**Published:** 2016-10-13

**Authors:** Zhe Shen, Stefan Munker, Boyan Zhou, Lin Li, Chaohui Yu, Youming Li

**Affiliations:** 1 Department of Gastroenterology, The First Affiliated Hospital, College of Medicine, Zhejiang University, 310003 Hangzhou, China; 2 Molecular Hepatology-Alcohol Associated Diseases, II. Medical Clinic Faculty of Medicine at Mannheim, University of Heidelberg, 68167 Mannheim, Germany; 3 Department of Biostatistics and Computational Biology, School of Life Sciences, Fudan University, Shanghai 200433, China

## Abstract

Magnetic resonance cholangiopancreatography (MRCP), MRCP after secretin stimulation (S-MRCP) and endoscopic ultrasonography (EUS) are all selected to diagnose pancreas divisum. However, the accuracies of three diagnosis remain unclear. The aim is to address the diagnostic accuracies of MRCP, S-MRCP and EUS on pancreas divisum. We searched PubMed, MEDLINE and EMBASE databases from inception to January, 2015. Of the 536 citations retrieved, 16 studies were included. For MRCP diagnosis on pancreas divisum, the area under the hierarchical summary receiver-operating characteristic (HSROC) curve was 0.90 (95% confidence interval [CI] 0.87 to 0.92), and for S-MRCP and EUS, 0.99 (95% CI 0.97 to 0.99) and 0.97 (95% CI 0.96 to 0.98). Sensitivity and specificity for MRCP were 0.59 (95% CI 0.45 to 0.71) and 0.99 (95% CI 0.96 to 1.00); for S-MRCP, 0.83 (95% CI 0.66 to 0.92) and 0.99 (95% CI 0.96 to 1.00); for EUS, 0.85 (95% CI 0.67 to 0.94) and 0.97 (95% CI 0.90 to 0.99). Comprehensive comparison of three diagnostic techniques to pancreas divisum, S-MRCP was more reliable than MRCP and EUS on the effect of the diagnostic test.

Pancreas divisum is the most common congenital anomaly of pancreatic anatomy, which is associated with acute or chronic pancreatitis^1^,^2^. The anomaly is the result of the absence of fusion between the ventral and dorsal pancreatic ducts, its prevalence is 5% to 14% in the general population^3^,^4^.

Generally, endoscopic retrograde cholangiopancreatography (ERCP) is considered as the criterion for diagnosing pancreas divisum, and it is commonly used as the conventional option for diagnosing pancreas divisum^5^. However, ERCP is an invasive diagnostic method which is associated with possible serious consequences^6^,^7^. Magnetic resonance cholangiopancreatography (MRCP) is non-invasive diagnostic technique that examines the biliary and pancreatic ducts actually^8^,^9^. Secretin could increase the volume of ductal fluid and the secretions by the exocrine pancreas^10^, so MRCP after secretin stimulation (S-MRCP) could improve the visualization of pancreatic ducts and enable the assessment of exocrine function of pancreas^11^,^12^,^13^. Endoscopic ultrasonography (EUS) is a minimally invasive test that shows detailed imaging of the pancreatic and ductal system^14^,^15^. Therefore, MRCP, S-MRCP and EUS are all selected to diagnose pancreas divisum. However, the accuracies and differences of the three diagnostic tests have been in conflict with each other and remain unclear until now. The aim of this study is to conduct a systematic review and meta-analysis to address the diagnostic accuracies of MRCP, S-MRCP and EUS in the detection of pancreas divisum.

## Results

### Eligible studies

**Figure 1:**
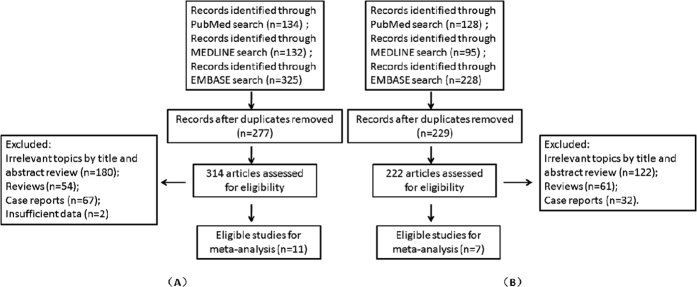
Flow diagram of the strategy and selected studies for MRCP (**A**) and EUS (**B**).

Of totally 314 unique studies that were retrieved by using the key word “MRCP”, 180 were excluded duing to irrelevant topics, 54 were excluded for reviews, 67 were excluded as case reports and 2 were excluded duing to insufficient data. Eventually, 11 studies fulfilled the inclusion criteria and were included in meta-analysis of MRCP^10^,^16^,^17^,^18^,^19^,^20^,^21^,^22^,^23^,^24^,^25^ (Fig. 1A). Of totally 222 unique studies that were retrieved by using the key word “EUS”, 122 were excluded duing to irrelevant topics, 61 were excluded as reviews and 32 were excluded as case reports. Eventually, 7 studies met the inclusion criteria and were therefore included in meta-analysis of EUS^16^,^19^,^26^,^27^,^28^,^29^,^30^ (Fig. 1B).

### Study characteristics

The 11 studies on the diagnosis of pancreas divisum by MRCP included 10 studies of totally 856 patients treated with MRCP and 5 studies of totally 625 patients treated with S-MRCP. The characteristics of the included studies are listed in Table 1, respectively. The 7 studies on the diagnosis of pancreas divisum by EUS pertained to a total of 470 patients. The characteristics of the included studies are also listed in Table 1.

### Quality assessment

Each of the included studies needed to be made quality assessment. The detailed results of the assessments are shown in Table 2. Generally, the included studies met most of the quality criteria and were therefore labeled as high quality. Certainly, no clear items were mentioned in many studies.

### Diagnostic performances of three techniques

**Figure 2:**
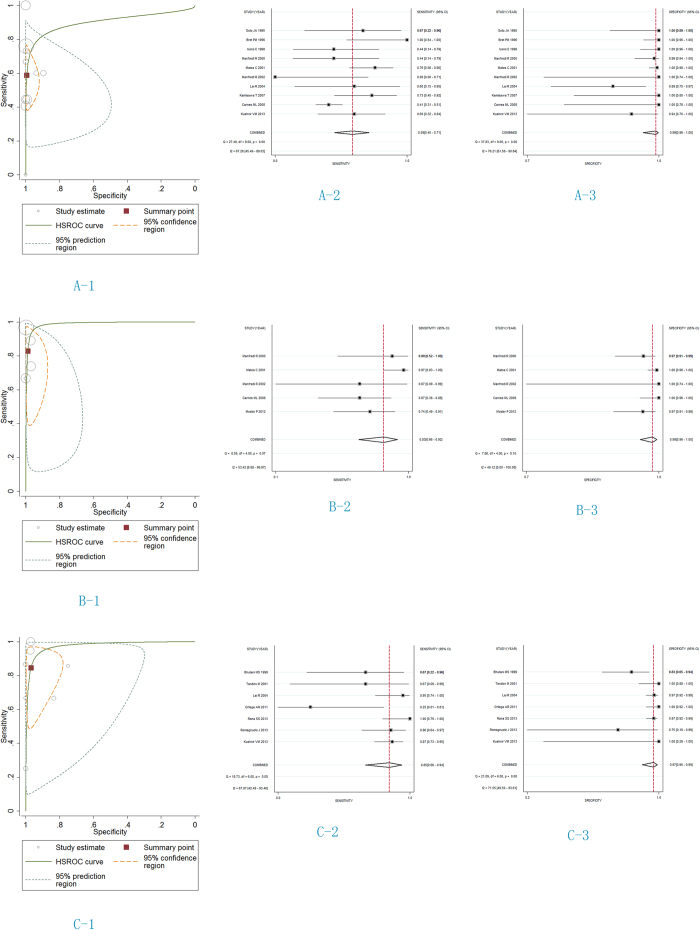
HSROC curves and forest plots for the diagnostic performance of MRCP, S-MRCP and EUS to diagnose pancreas divisum. The size of the circles shows the weighting of each study. For MRCP (**A-1**), the area under the HSROC curve was 0.90 (95% CI 0.87 to 0.92), the sensitivity (**A-2**) and specificity (**A-3**) were 0. 59 (95% CI 0.45 to 0.71) and 0.99 (95% CI 0.96 to 1.00). For S-MRCP (B), the area under the HSROC curve was 0.99 (95% CI 0.97 to 0.99), the sensitivity (**B-2**) and specificity (**B-3**) were 0. 83 (95% CI 0.66 to 0.92) and 0.99 (95% CI 0.96 to 1.00). For EUS (C), the area under the HSROC curve was 0.97 (95% CI 0.96 to 0.98), the sensitivity (**C-2**) and specificity (**C-3**) were 0. 85 (95% CI 0.66 to 0.94) and 0.97 (95% CI 0.90 to 0.99).

The diagnostic performance of MRCP on pancreas divisum showed the area under the HSROC curve was 0.90 (95% confidence interval [CI] 0.87 to 0.92) (Fig. 2A-1). S-MRCP diagnosis on pancreas divisum showed highly accurate diagnostic performance, with the area under the HSROC curve being 0.99 (95% CI 0.97 to 0.99) (Fig. 2B-1). There was also highly accurate diagnostic performance with EUS, with the area under the HSROC curve being 0.97 (95% CI 0.96 to 0.98) (Fig. 2C-1).

The main results are listed in Fig. 2 and Table 3. In ten studies on MRCP, the sensitivity and specificity for MRCP diagnosis were 0.59 (95% CI 0.45 to 0.71) and 0.99 (95% CI 0.96 to 1.00), respectively; positive likelihood ratio (LR+) and negative likelihood ratio (LR−) were 87.83 (95% CI 15.25 to 505.81) and 0.42 (95% CI 0.30 to 0.58); the diagnostic odds ratio (OR) was 211.33 (95% CI 32.14, 1389.76). In five studies on S-MRCP, the sensitivity and specificity for S-MRCP diagnosis were 0.83 (95% CI 0.66 to 0.92) and 0.99 (95% CI 0.96 to 1.00), respectively; LR+ and LR− were 65.48 (95% CI 20.85 to 205.71) and 0.17 (95% CI 0.08 to 0.37); the diagnostic OR was 376.89 (95% CI 75.02, 1893.37). In seven studies on EUS, the sensitivity and specificity for EUS diagnosis were 0.85 (95% CI 0.67 to 0.94) and 0.97 (95% CI 0.90 to 0.99), respectively; - LR+ and LR− were 26.80 (95% CI 8.05 to 89.27) and 0.16 (95% CI 0.07 to 0.38), respectively; the diagnostic OR was 167.89 (36.96, 762.69).

### Sensitivity analysis

We systematically removed one portion of data randomly and recalculated the Log OR for the remaining studies of MRCP, S-MRCP and EUS, respectively (Table 4). The three results were similar, indicating that no single data could alone significantly influence the combined analysis of MRCP, S-MRCP and EUS.

### Bias Diagnostics

**Figure 3:**
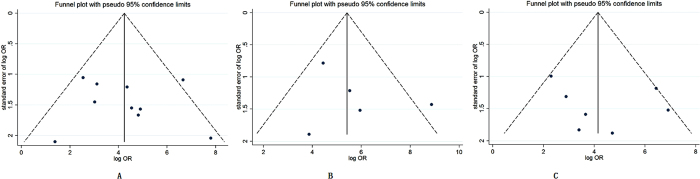
Funnel plot to evaluate publication bias of selected studies for MRCP (**A**) S-MRCP (**B**) and EUS (**C**).

No significant publication bias for the three techniques on MRCP, S-MRCP and EUS was detected through the Begg-Mazumdar test and the Harbord-Egger test (all *P* > 0.05). No visual publication bias was found in the funnel plot for MRCP, S-MRCP and EUS, respectively (Fig. 3).

## Discussion

The finding of the meta-analysis suggested that the diagnosis of pancreas divisum using whichever technique such as MRCP, S-MRCP or EUS, has a high diagnostic performance. The lowest area under HSROC curve in the three techniques reached 0.90. Pancreas divisum is the congenital anomaly in which the dorsal and ventral pancreatic glands drain separately into the duodenum^31^. Although ERCP has been proved to have high diagnostic accuracy in the excellent description of the biliary and pancreatic ductal system and has been accepted as the gold standard for diagnosing pancreas divisum^31^,^32^, it is an invasive procedure and represents a certain degree of complications^33^. MRCP is non-invasive diagnostic technique and EUS is a minimally invasive test, which both give negligible complications. The two techniques have been widely used to investigate the pancreatic and ductal system in clinical practice. However, the specific accuracies of diagnosing pancreas divisum by MRCP, S-MRCP and EUS remain unclear. The number of related studies on the diagnosis of pancreas divisum with MRCP or EUS is increasing year by year, so it is the right time to combine these studies together and to compare the accuracies of different techniques on diagnosing pancreas divisum.

MRCP is one of the first choices for examining biliary and pancreatic ductal system in a non-invasive way. Secretin is used to strengthen the visualization of the pancreatic duct at MRCP, and its working mechanism is mainly to stimulate pancreas to secrete fluid and bicarbonate and to improve the visualization of the pancreatic duct^10^,^21^. Actually, this meta-analysis showed the area under HSROC curve for S-MRCP (0.99, 95% CI, 0.97–0.99) was larger than that for MRCP (0.90, 95% CI, 0.87–0.92); the diagnostic OR for S-MRCP (376.89, 95% CI, 75.02–1893.37) was larger than that for MRCP (211.33, 95% CI, 32.14–1389.76). This suggested that S-MRCP was superior to MRCP in terms of the effect of the diagnostic test. If the likelihood ratios are greater than 10 but less than 0.1, the results will suggest strong evidence for meeting a diagnosis in or out, respectively. The meta-analysis showed the pooled likelihood ratios LR+ and LR−for S-MRCP were 65.48 (95% CI, 20.85–205.71) and 0.17 (95% CI, 0.08–0.37); the pooled likelihood ratios LR+ and LR−for MRCP were 87.83 (95% CI, 15.25–505.81) and 0.42 (95% CI, 0.30–0.58). Combining these results together, we concluded that S-MRCP was more reliable than MRCP on diagnosing pancreas divisum.

EUS has definite advantages over other options in evaluating biliary and pancreatic ductal system, and it is less invasive than ERCP. EUS could achieve detailed imaging of the biliary and pancreatic ductal system without injecting contrast into these ducts. Therefore, pancreas divisum could also be detected by minimally invasive techniques like EUS, but EUS could obviate the associated risks like ERCP^34^. This meta-analysis showed the area under HSROC curve for EUS was 0.97 (95% CI, 0.96–0.98), suggesting that EUS was superior to MRCP but slightly inferior to S-MRCP in terms of the effect of the diagnostic test. Additionally, the meta-analysis showed the pooled likelihood ratios LR+ and LR− for EUS were 26.80 (95% CI, 8.05–89.27) and 0.16 (95% CI, 0.07–0.38), respectively. However, the diagnostic OR for S-MRCP (376.89, 95% CI, 75.02–1893.37) was larger than that for EUS (167.89, 95% CI, 36.06–762.69). This suggested that EUS was more reliable than MRCP in diagnosing pancreas divisum, but it was inferior to S-MRCP.

There are several limitations about the present meta-analysis of literature data. First, there are 10 studies of totally 856 patients for MRCP diagnosis of pancreas divisum, so the MRCP diagnosis involves twice as many studies as S-MRCP has, and nearly twice as many patients as EUS has. So the number of studies and patients are different among the three methods. Second, ERCP is considered as the gold standard for pancreas divisum, but fails to cannulate a certain proportion. We didn’t account the part of ERCP failure rates in this meta-analysis. Additionally, therapeutic potential of ERCP could add perspective, it’s pity that there were no further explanations in these studies. Third, recurrent pancreatitis or chronic pancreatitis may destroy or alter pancreatic ducts, there were no clear explanations in these studies, which could result in a certain bias in the meta-analysis. Fourth, a large portion of studies are observational and nonrandomized controlled studies. These suggest that there are some confounding factors influencing the meta-analysis. Fifth, S-MRCP and EUS are relatively newer technology than MRCP, publications may favor positive reports, so each of the included studies needed to be made quality assessment to avoid the potential bias as much as possible. The results are limited by the quality and quantity of the present data. Certainly, no significant publication bias for the three techniques was detected through the Begg-Mazumdar test and the Harbord-Egger test, and the results were further confirmed by the funnel plot. A large part of studies and patients were performed in tertiary centers, but it was not clear whether gastrointestinal radiologists or general radiologists reviewed the results. Therefore, there is expertise bias in this study because of differnent hospitals and different experienced doctors.

In conclusion, comprehensive comparison of three diagnostic techniques to pancreas divisum, S-MRCP was more reliable than MRCP and EUS on the effect of the diagnostic test.

## Methods

### Literature retrieval strategy and selection

We conducted an online literature retrieval or search from PubMed, MEDLINE and EMBASE databases from inception to January, 2015. The key words of the heading used in the search included “pancreas divisum” AND (“cholangiopancreatography, magnetic resonance” OR “magnetic Resonance Imaging” OR “magnetic resonance cholangiopancreatography” OR MRCP); “pancreas divisum” AND (“endoscopic ultrasound” OR EUS OR ultrasound OR endosonography). No restrictions were imposed on the choice of languages. The titles and abstracts that included these terms were in detail for potential inclusion. The full text of the remaining articles including the references was ascertained to have contained related information.

We included studies evaluating the detection of pancreas divisum with MRCP and/or S-MRCP and/or EUS as the reference standard. The studies could be represented by 2 × 2 tables with true-positive, false-negative, false-positive and true-negative values. We excluded those studies that did not involve the detection of pancreas divisum, those with insufficient data, as well as those primarily designed as the reviews, editorials, case reports or meta-analysis. We discussed and resolved disagreement between the investigators who evaluated the detection of pancreas divisum.

### Data extraction

Data were seperately extracted and printed in standardized paper forms. The following data were collected for all studies: study design, period of study/year of publication, country, number of patients, mean age, male to female ratio, criteria for pancreas divisum, tertiary center and main outcomes reported.

### Assessment of study quality

The Quality Assessment of Diagnostic Accuracy Studies tool was used to evaluate the quality of the studies. We rated the quality of key study design characteristics of a total of 14 items^35^. The 14 items were rated to evaluate the quality of key study design characteristics in this analysis.

### Statistical analysis

Meta-analysis for diagnosis of pancreas divisum was performed under a linear mixed model approach to calculate summary estimates of sensitivity, specificity, LR+, LR−, and diagnostic OR all of which were then fitted into a HSROC curve^36^,^37^. We assessed the publication bias qualitatively and quantitatively by funnel plots and bias indicators, including by the Begg-Mazumdar test and the Harbord-Egger test^38^,^39^. We performed sensitivity analysis to calculate whether any single study was contributing undue weighting to the analysis. We removed one portion of study data and checked the pooled results to see whether there was any significant change in test performance. We used Stata V.12 to perform the calculations.

## Additional Information

**How to cite this article**: Shen, Z. *et al*. The Accuracies of Diagnosing Pancreas Divisum by Magnetic Resonance Cholangiopancreatography and Endoscopic Ultrasound: A Systematic Review and Meta-analysis. *Sci. Rep.* **6**, 35389; doi: 10.1038/srep35389 (2016).

## Figures and Tables

**Table 1:** Study design and statistical characteristics of included studies on MRCP, S-MRCP and EUS.

Author	Year	Location	Patients	Mean age (yr)	Men/Female	Reference standard	Tertiary center	TP	FP	FN	TN	Sens (%)	Spec (%)
*MRCP*													
Kushnir VM *et al*.^16^	2013	USA	31	53	19/12	ERCP	Yes	9	1	6	15	60.0	93.8
Carnes ML *et al*.^17^	2008	USA	111	60	84/27	ERCP	Partly	39	0	57	15	40.6	100
Kamisawa T *et al*.^18^	2007	Japan	32	NS	NS	ERCP	Yes	11	0	4	17	73.3	100
Lai R *et al*.^19^	2004	USA	43	51	NS	ERCP	Yes	3	4	2	34	60	89.5
Manfredi R *et al*.^20^	2002	Italy	15	11.3	NS	ERCP	Yes	0	0	3	12	0	100
Matos C *et al*.^21^	2001	Belgium	279	51.4	171/108	ERCP	Yes	22	1	7	249	75.9	99.6
Manfredi R *et al*.^10^	2000	Italy	107	48	56/51	ERCP	Yes	4	1	5	97	44.4	99.0
Ueno E *et al*.^22^	1998	Japan	93	NS	NS	ERCP	Yes	4	0	5	84	44.4	100
Bret PM *et al*.^23^	1996	Canada	108	NS	NS	ERCP	Yes	6	0	0	102	100	100
Soto JA *et al*.^24^	1995	USA	37	53	14/23	ERCP	Yes	4	0	2	31	66.7	100
*S-MRCP*													
Mosler P *et al*.^25^	2012	USA	113	47.1	54/59	ERCP	Yes	14	3	5	91	73.7	96.8
Carnes ML *et al*.^17^	2008	USA	111	60	84/27	ERCP	Yes	10	0	5	96	66.7	100
Manfredi R *et al*.^20^	2002	Italy	15	11.3	NS	ERCP	Yes	2	0	1	12	66.7	100
Matos C *et al*.^21^	2001	Belgium	279	51.4	171/108	ERCP	Yes	29	1	1	248	96.7	99.6
Manfredi R *et al*.^10^	2000	Italy	107	48	56/51	ERCP	Yes	8	3	1	95	88.9	96.9
*EUS*													
Kushnir VM *et al*.^16^	2013	USA	45	53.8	13/32	ERCP	Yes	39	0	6	3	86.7	100
Romagnuolo J *et al*.^26^	2013	USA	36	55	16/20	ERCP	Yes	18	1	3	3	85.7	75
Rana SS *et al*.^27^	2013	India	146	36.9	102/44	ERCP	Yes	16	4	0	126	100	96.9
Ortega AR *et al*.^28^	2011	Spain	49	58	24/25	Unclear	Yes	1	0	3	45	25	100
Lai R *et al*.^19^	2004	USA	127	51	NS	ERCP	Yes	18	3	1	105	94.7	97.2
Tandon M *et al*.^29^	2001	USA	31	48.8	12/19	ERCP	Partly	2	0	1	28	66.7	100
Bhutani MS *et al*.^30^	1999	USA	36	NS	NS	ERCP	Yes	4	5	2	25	66.7	83.3

**Table 2:** Quality Assessment Tool for Diagnostic Accuracy Systematic Review to evaluate the quality of selected studies.

Study	C1	C2	C3	C4	C5	C6	C7	C8	C9	C10	C11	C12	C13	C14
Kushnir VM *et al*.^16^	√	√	√	√	√	√	√	√	√	?	?	√	√	√
Romagnuolo J *et al*.^26^	√	√	√	√	×	×	√	√	×	√	√	√	√	×
Rana SS *et al*.^27^	√	√	√	√	×	×	√	√	√	×	×	√	√	√
Mosler P *et al*.^25^	√	√	√	√	√	√	√	√	√	?	?	√	√	√
Ortega AR *et al*.^28^	√	√	?	√	×	√	√	√	×	×	×	√	√	√
Carnes ML *et al*.^17^	√	√	×	√√	×	×	√	√	√	?	?	√	√	√
Kamisawa T *et al*.^18^	√	√	√	√	√	√	√	√	√	×	×	√	√	√
Lai R *et al*.^19^	√	√	√	√	√	√	√	√	√	×	×	√	√	√
Manfredi R *et al*.^20^	√	√	√	√	√	√	√	√	√	?	?	√	√	√
Matos C *et al*.^21^	√	√	√	√	√	√	√	√	√	×	×	√	√	√
Tandon M *et al*.^29^	√	√	√	√	×	×	√	√	×	×	×	√	√	√
Manfredi R *et al*.^10^	√	√	×	√	×	×	√	√	√	×	×	√	√	√
Bhutani MS *et al*.^30^	√	√	√	√	×	×	√	√	√	×	×	√	√	√
Ueno E *et al*.^22^	√	√	√	√	√	√	√	√	√	?	√	√	√	√
Bret PM *et al*.^23^	√	√	√	√	√	√	√	√	√	?	√	√	√	√
Soto JA *et al*.^24^	√	√	√	√	√	√	√	√	√	?	√	√	√	√

C1: Patient spectrum representative?

C2: Selection criteria described?

C3: Reference standard appropriate?

C4: Time between tests appropriate?

C5: Uniform verification by reference standard?

C6: Same reference test used?

C7: Reference standard independent?

C8: Index test described adequately?

C9: Reference standard described adequately?

C10: Blinding to reference standard results?

C11: Blinding to index test results?

C12: Appropriate clinical data available?

C13: Uninterpretable data reported?

C14: Withdrawals explained?

**Table 3:** Diagnostic accuracy of pancreas divisum diagnosis with MRCP, S-MRCP and EUS, respectively.

Study characteristics	No. of studies	Likelihood ratio (95% CI)		Area under HSROC curve (95% CI)	Diagnostic OR (95% CI)
		LR+	LR−		
MRCP	10	87.83(15.25, 505.81)	0.42(0.30, 0.58)	0.90(0.87, 0.92)	211.33(32.14, 1389.76)
S-MRCP	5	65.48(20.85, 205.71)	0.17(0.08, 0.37)	0.99(0.97, 0.99)	376.89(75.02, 1893.37)
EUS	7	26.80(8.05, 89.27)	0.16(0.07, 0.38)	0.97(0.96, 0.98)	167.89(36.96, 762.69)

**Table 4:** Sensitivity Analysis when systematically removing 1 data randomly.

Study characteristics	LogOR(combined)	Max LogOR	Min LogOR
MRCP	4.26(3.16,5.37)	4.55(3.41,5.68)	3.80(2.83,4.76)
S-MRCP	5.68(4.05,7.31)	6.20(4.26,8.15)	4.85(3.72,5.98)
EUS	4.27(2.80,5.74)	4.78(3.34,6.22)	3.75(2.36,5.14)

Log is Ln in this study.

LogOR(combined) is the data for all studies; Max LogOR and Min LogOR are the largest and smallest data when systematically removing 1 data, respectively.
